# MOVING: Motivation-Oriented interVention study for the elderly IN Greifswald: study protocol for a randomized controlled trial

**DOI:** 10.1186/s13063-017-2425-2

**Published:** 2018-01-22

**Authors:** Fabian Kleinke, Thea Schwaneberg, Franziska Weymar, Peter Penndorf, Sabina Ulbricht, Kristin Lehnert, Marcus Dörr, Wolfgang Hoffmann, Neeltje van den Berg

**Affiliations:** 1 Institute for Community Medicine, Section Epidemiology of Health Care and Community Health, University Medicine Greifswald, Greifswald, Germany; 2 DZHK (German Centre for Cardiovascular Research), partner site Greifswald, Greifswald, Germany; 3 Institute of Social Medicine and Prevention, University Medicine Greifswald, Greifswald, Germany; 4 Department of Internal Medicine B, University Medicine Greifswald, Greifswald, Germany

**Keywords:** Physical activity, Sedentary behavior, Accelerometry, Elderly individuals (people), motivation, Behavior change, Intervention, RCT

## Abstract

**Background:**

Cardiovascular diseases (CVD) are the leading cause of mortality. In 2014, they were responsible for 38.9% of all causes of death in Germany. One major risk factor for CVD is a lack of physical activity (PA). A health-promoting lifestyle including regular PA and minimizing sitting time (ST) in daily life is a central preventive measure. Previous studies have shown that PA decreases in older age; 2.4–29% of the people aged over 60 years achieve the World Health Organization recommendations. This age group spends on average 9.4 h per day in sedentary activities.

To increase PA and decrease ST, a low-threshold intervention, consisting of individualized feedback letters based on objectively measured data of PA and ST, was developed. The research question is: Do individual feedback letters, based on accelerometer data, have a positive effect on PA and ST?

**Methods/design:**

MOVING is a two-arm, randomized controlled trial. Inclusion criteria are age ≥ 65 years and the ability to be physically active. Exclusion criteria are the permanent use of a wheelchair and simultaneous participation in another study on PA.

At baseline participants who give informed consent will receive general information and recommendations about the positive effects of regular PA and less ST. Participants of both groups will receive an accelerometer device, which records PA and ST over a period of seven consecutive days following by a randomization. Participants in the intervention group will receive automatically generated, individualized feedback letters by mail based on their PA and ST at baseline and at 3-month follow-up. Further follow-up examinations will be carried out at 6 and 12 months.

The primary outcome is the increase of PA and the reduction of ST after 6 months in the intervention group compared to the control group.

**Discussion:**

The goal of the study is to examine the effects of a simple feedback intervention on PA and ST in elderly people. We aim to achieve an effect of 20% increase in moderate-to-vigorous physical activity (MVPA). The intervention may have the potential to decrease crucial cardiovascular risk factors and, therefore, contribute to prevention of CVD.

**Trial registration:**

German Clinical Trials Register, ID: DRKS00010410. Registered on 17 May 2017.

**Electronic supplementary material:**

The online version of this article (10.1186/s13063-017-2425-2) contains supplementary material, which is available to authorized users.

## Background

Older age is associated with increasing risk for chronic diseases and multimorbidity [1–3]. The prevalence of cardiovascular diseases (CVD), including coronary heart disease (CHD) and stroke, increases with age [4]. In 2014, CVD were the leading cause of mortality [5] and responsible for 38.9% of all causes of death in Germany [6]. CVD produce substantial disabilities and reduce well-being [7, 8]. Therefore, older people are a relevant target group for effective and practicable strategies for CVD prevention [9].

According to the World Health Organization (WHO), lack of PA is an important risk factor for mortality [10] and is associated with a series of non-communicable diseases (NCD), e.g., type 2 diabetes and a number of cancers (e.g., colon cancer and breast cancer) [7]. Several studies have shown that a health-promoting lifestyle, including promotion of PA and reduction of ST, is correlated with improved overall health status [7, 11]. Furthermore, regular PA is a resource for physical and mental health and reduces risks of obesity and musculoskeletal system problems. Regular PA significantly decreases overall mortality by 22–34% and CVD mortality by 27–35% [12].

In general, international PA guidelines do not recommend that elderly people do less PA than younger adults [13–15]. The WHO recommends 150 min of moderate or 75 min of vigorous PA, or a corresponding combination of weekly PA for both for younger adults (aged 18–64 years) and older people aged over 65 years. PA should be done in time periods (bouts) of at least 10 min [14].

Recent academic literature points out that PA decreases in older age [16–18] while the amount of ST increases to high levels (about an average of 60% ST of waking time) [19, 20]. In Germany, 19.3% of men and 16.8% of women aged 60–69 years achieved the WHO recommendations. In the age group 70–79 years the amount of PA declines to 16.5% of men and 11.0% of women [10]. A study from the United Kingdom showed that 19% of men and 14% of women aged 65–74 years reach the recommended 30 min of moderate PA at least five times a week [7]. Data from Norway showed similar results among older people: 29% of men and 25% of women aged 65–69 years met the Norwegian PA recommendations (minimum of 30 min of daily PA of moderate intensity). In the age group 80–85 years the amount of PA declines; 7.1% of women and 3% of men reached the recommendations [9].

The National Health and Nutrition Survey (NHANES, USA) showed that 2.4% of people aged over 60 years achieved WHO recommendations for PA [21]. This age group spends on average 8.4 h (60–69) and 9.3 h (70–85) per day in sedentary activities (measured by accelerometer) which correspondents up to 70% of their wake time [19]. A systematic review of adults aged 60 years and older showed similar results: older people spend an average of 9.4 h doing sedentary activities which corresponds to 65–80% of their waking day. It is particularly striking that estimated self-reported ST is lower than objectively measured: on average 5.3 h per day [22]. In the aforementioned Norwegian study participants aged 65–85 years spend 66% of the day in ST, 24% doing low-intensity PA and 3% doing moderate-to-vigorous physical activity (MVPA) [9].

The variation in the data can be explained by the fact that studies are conducted in different settings and the definition of elderly people varies. Furthermore, there are differences in the assessment of PA data. Some studies are based on measured accelerometer data, others on self-reported data. PA assessment based on self-reporting methods may not quantify PA levels correctly [7]. The use of accelerometer devices for objective assessment of PA allows a valid and reliable record of activity intensity, frequency, and duration which may improve our understanding of PA in the elderly [7, 23].

Individualized feedback on accelerometer measurements can help to increase PA and reduce ST [24–26]. Interventions in promoting PA should consider behavioral-change aspects. A systematic review regarding interventions to promote walking identified two general characteristics of effective interventions. These aspects are *targeting* and *tailoring* and involve addressing participants’ requirements or circumstances [27].

Harris et al. showed statistically significant positive effects in the age group 60–75 years on the amount of PA (average daily step-counts and weekly MVPA in ≥ 10-min bouts) [26]. This intervention included four primary care nurse consultations during a period of 3 months. Participants received feedback on the basis of pedometer step-counts, accelerometer PA intensity, and an individual PA diary and plan. Furthermore, a meta-analyses regarding the impact of pedometer-based physical activity interventions showed that the use of pedometers has a moderate positive effect on PA (an average increase of 2000 steps per day [28]. A systematic review of the use of pedometer interventions showed similar results; participants increased their PA by 2491 steps per day in comparison to the control group [29].

Most previous studies have been characterized by rather complex interventions and also included feedback of PA and ST results, personal consultations, and individual target agreements. A translation of such interventions into real life may be difficult due to considerable costs and effort to reach a large number of people.

Hence, we developed a low-threshold intervention consisting of automatically retrieved, individualized feedback letters, based on accelerometer data. In a randomized controlled trial, the effect of this feedback intervention on the amount of PA and ST will be examined prospectively.

The study protocol follows the Standard Protocol Items: Recommendations for Interventional Trials (SPIRIT) guidelines Additional file 1 [30].

### Research objectives

The primary objective of the MOVING study is to examine the effects of an increase of PA and a reduction of ST after 6 months in the intervention group compared to the control group as assessed by accelerometry.

Secondary outcomes are whether there is an improvement of blood pressure, a reduction in weight and waist circumference as well as an increase of self-reported time doing PA, self-reported ST, and self-reported time doing PA as well as the effect of feedback letters on self-efficacy after 6 and 12 months.

Additionally, the impact of personal feedback letters on lifestyle (household, gardening, free time, and sport activities), quality of life and type of PA will be evaluated by questionnaire in the intervention group compared to control group after 6 and 12 months.

## Methods/design

The study region is Western Pomerania, a rural area in the Northeast of Germany.

**Fig. 1:**
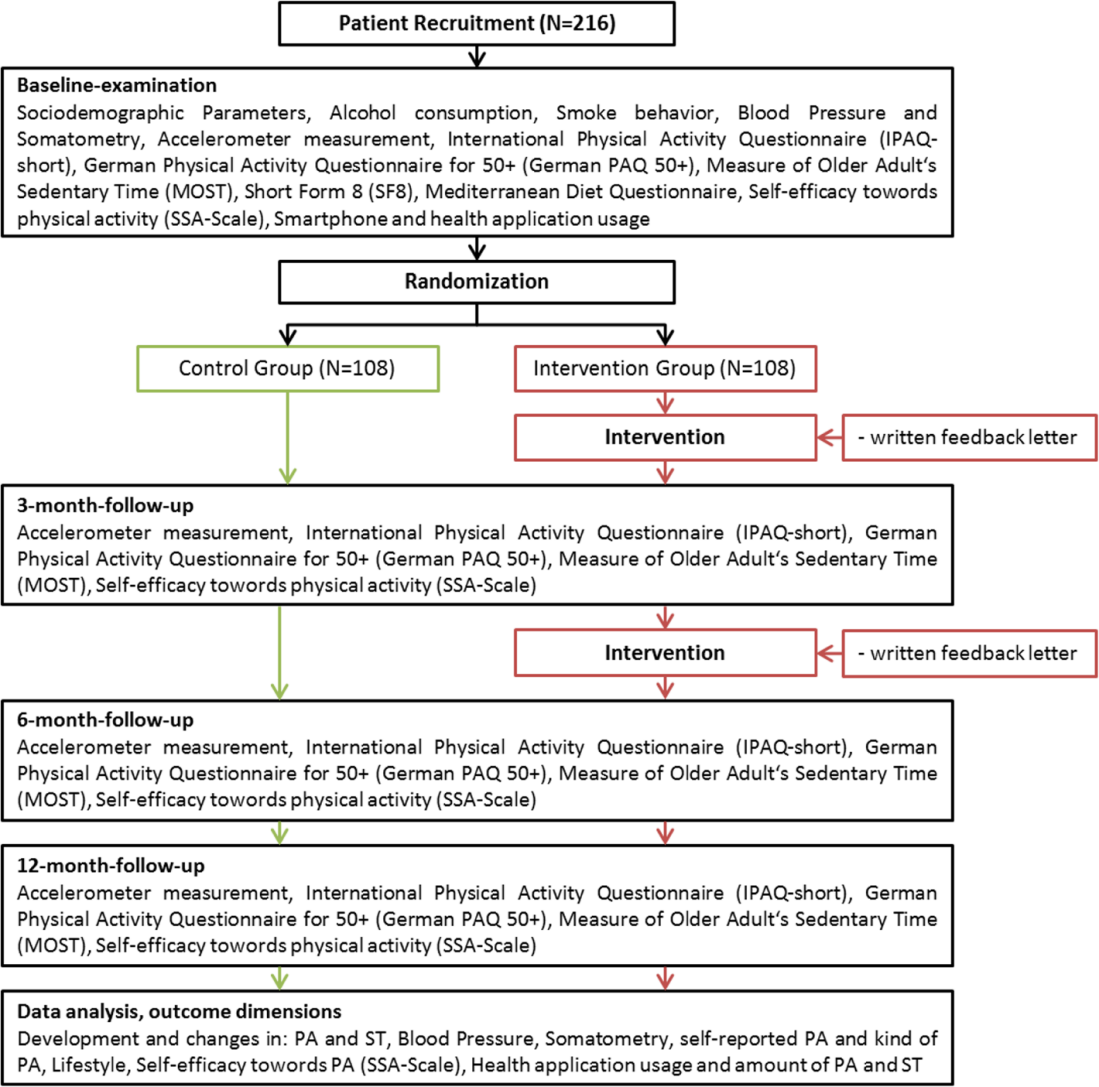
Flow chart of the study

MOVING (Motivation-Oriented interVention study for the elderly IN Greifswald) is a two-arm, randomized controlled trial consisting of participant screening, baseline examination, randomization, intervention, and follow-up examinations at 3, 6, and 12 months after baseline. The flow chart of the study is shown in Fig. 1.

### Study population

The study population consists of elderly people sampled from the general population who meet the following inclusion criteria:

1. Age ≥ 65 years
2. The possibility of being physically active in daily life

Exclusion criteria are:

1. Permanent use of a wheelchair (no ability to walk independently)
2. Simultaneous participation in other studies including PA or ST
3. Not accessible by telephone or cell phone (necessary for screening)
4. Fulfillment of the WHO recommendations for PA (self-report) for people aged ≥ 65 years at baseline

### Recruitment and screening

Participants will be recruited in three ways:

1. Re-contacting participants of a previous study.
  Participants have given their consent to re-contacting for scientific projects
2. Active recruitment in general medical practices (GP)
3. Recruitment of volunteers (e.g., in medical practices, hospitals, meeting centers, and via flyers and posters)

Potential participants contact the study staff by telephone or by use of a reply card with contact information. A short telephone screening of potential study participants is performed to assess the inclusion and exclusion criteria. The data will be documented in a documentation system based on electronic Case Report Forms (eCRFs). For participants who meet the inclusion criteria, an appointment for the baseline examination at the examination center of the German Centre for Cardiovascular Research (DZHK) Greifswald is made.

### Baseline examination

At the examination center, the participants are informed in detail about the study and invited to give written informed consent. Upon consent all receive a baseline examination consisting of blood pressure assessment and somatometry data (body weight, waist and hip circumference). All measurements are carried out by trained and certified study staff according to standard operating procedures (SOP).

In addition, all participants receive paper-pencil questionnaires to assess sociodemographics, general health, and actual PA behavior. To asses health status and quality of life the Short Form 8 (SF-8) questionnaire (German version) is used, which consists of eight scales addressing mental and physical health [31].

Furthermore, all study participants will receive the SSA scale (self-efficacy towards physical exercise) to assess each participant’s level of self-efficacy (twelve items which can be summed to a total score in which higher results indicate higher self-efficacy towards PA) [32]. To assess dietary habits, the Mediterranean Diet questionnaire (14 items, score ranging from 0 to 14) will be used [33]. Alcohol consumption will be assessed by using two items from the Alcohol Use Disorders Identification Test (AUDIT) [34].

After answering the questionnaires, all participants receive an accelerometer device (ActiGraph wGT3X-BT, Pensacola, FL, USA). The validated three-axis accelerometer device captures and records continuously PA and ST. Participants are instructed to wear the device during a period of seven consecutive days on the right hip and to remove it only for water-based activities (e.g., showering and swimming) as well as at bedtime. Data will be recorded at a sampling frequency of 30 Hz. Furthermore, participants are asked to document their activities in a semi-standardized daily protocol and to answer several paper-based questionnaires about PA and ST after the 7 days of accelerometer use at home.

**Fig. 2:**
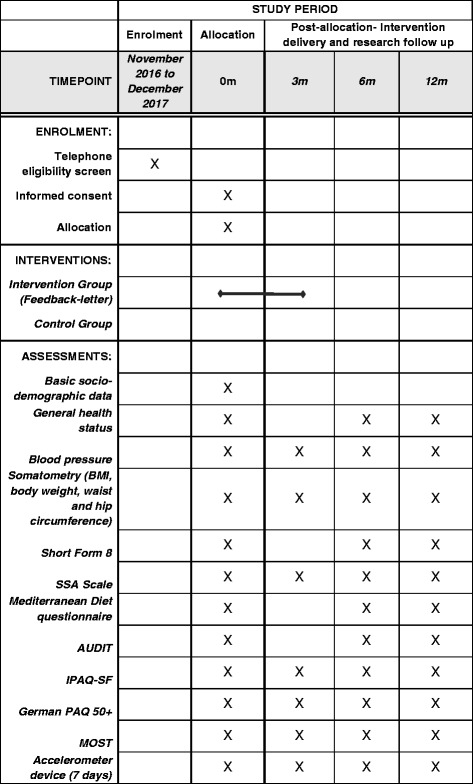
Schedule of enrollment, interventions, and assessments

To assess self-reported PA, the International Physical Activity Questionnaire short form (IPAQ-SF), German version is used. The IPAQ consists of seven items, addressing intensity and duration of PA in daily life over the last 7 days by self-report [35]. Additionally, the German Physical Activity Questionnaire for 50+ (German PAQ 50+) is conducted to assess type and duration of PA in daily life by self-report [36]. Sedentary behavior is captured by the Measure of Older Adults’ Sedentary Time (MOST Questionnaire) in the German version, which consists of seven items [37] (Fig. 2).

At baseline all participants receive general information and recommendations about the positive effects of regular PA and less ST on the improvement of cardiovascular risk factors.

Therefore, all study subjects will receive age-appropriate literature from the Federal Centre for Health Education (BZgA) [38] and a chart from the Fonds Gesundes Österreich (FGÖ) [39]. Recommendations provided for PA are based on WHO guidelines for PA for people of age ≥ 65 years [14].

### Random allocation

After screening and accelerometry, participants will be randomized 1:1 in the intervention and the control group. The randomization will be conducted using an automated function in the documentation software.

### Study intervention

We developed a low-threshold intervention characterized by easy access to the intervention and a low effort for the target group. The intervention comprises two individualized feedback letters, automatically generated in R software (version 3.3.2, Lucent Technologies, Murray Hill, NJ, USA) from the study data base using the variables number of steps per day, time in minutes of MVPA, and sedentary time in min per day.

**Fig. 3:**
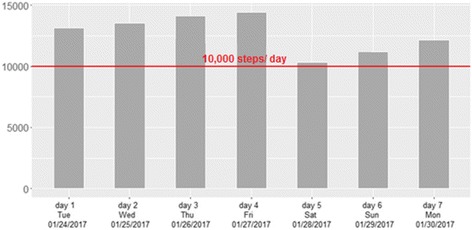
Graph of steps taken per day

Participants in the intervention group will receive a feedback letter by mail shortly after wearing the accelerometer after the baseline examination and after the 3-month follow-up. The feedback letters will contain personalized feedback based on accelerometry as well as ST behavior. PA and ST data will be depicted in three comprehensive graphs:

One graph will represent the number of steps per day that participants took over the 7 days wearing the accelerometer device (Fig. 3).

Therein, 10,000 steps per day will be marked as a red line. Ten thousand steps, has been chosen because this number is used in several prevention programs in Germany and is well known in the population [40, 41].

**Fig. 4:**
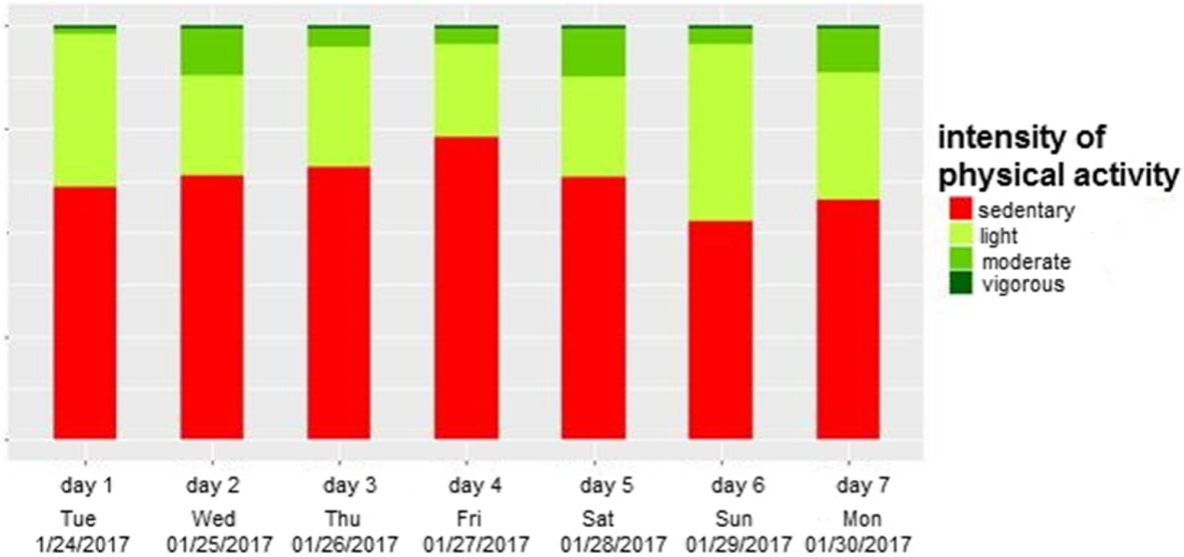
Graph intensity of physical activity (PA) per day

The second graph will constitute intensity of PA divided into colored bar charts per day from red for sedentary activities to dark green for vigorous activities (Fig. 4).

**Fig. 5:**
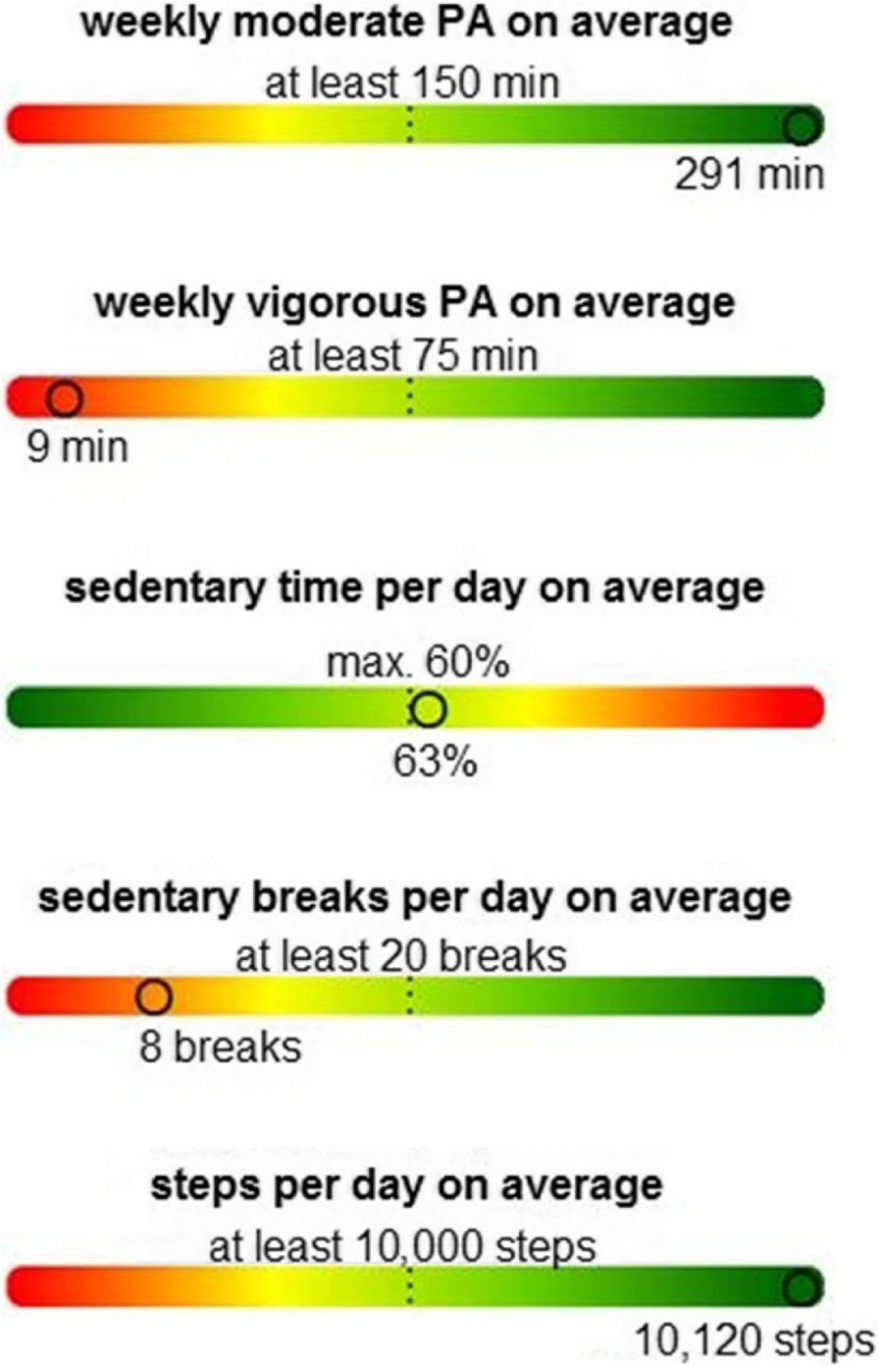
Graph of specific physical activity (PA) and sitting time (ST)

The third graph will show time doing moderate and vigorous activities per week, sitting time per day in percent as a weekly average, count of sit-to-stand transactions as a weekly average, and counts per day as a weekly average measure. To communicate these individual PA and ST measurements effectively to the participants, we will use a multidimensional representation using color bar charts (green represents a high PA level, red represents a low PA level) added with specific recommendations (Fig. 5).

Additionally, participants receive leaflets with age-appropriate recommendations for PA and ST at baseline. The leaflets are from the Federal Centre for Health Education.

### Follow-up

The follow-up examinations after 3, 6, and 12 months will be conducted in the DZHK examination center. Following examination, PA and ST will be objectively measured by the personal accelerometer. After a period of 7 days, participants bring back the accelerometer devices. In addition, study participants receive paper-based questionnaires as shown in the flow chart in Fig. 1.

### Power calculation

The sample size estimate was based on the amount of PA (mean time in min per day), measured in a comparable group of elderly people. In the study in question (Troiano et al.) the amount of PA was on average 15 min of combined PA (moderate and vigorous PA) per day [21]. We took these data as the starting point for the estimation of the required sample size. We assume that participants in the control group remain at approximately the same level while people in the intervention group increase their amount of PA by 20% [42], which corresponds to an increase from 15 to 18 min of PA per day after 6 months. To demonstrate this effect, a total number of 151 participants is needed (standard deviation 6.0 min, alpha = 0.05, power = 0.80). Assuming a loss to follow-up of about 30%, we will recruit 216 participants.

### Documentation, data storage, data security, and data protection

Participants’ sociodemographics (age, sex, education), lifestyle parameters (nutrition habits, smoking, and alcohol-drinking behavior) from the paper-based questionnaires (self-report) are documented using the Cardiff TeleForm system® (Electric Paper, Lüneburg, Germany). The questionnaires and the daily diary protocol contain 1-D barcodes to ensure anonymization. Somatometry data and blood pressure measurements will be documented in eCRFs in an IT-supported documentation system including automatic plausibility and completeness checks [43]. The documentation system is based on the concept of offline clients, each staff member in the examination center has individual login data [43].

After merging the data assessed in the examination center with the PA data from the accelerometer and the data from the paper-based questionnaires, all data will be stored in a central project data base. The data storage is managed according to current standards for data security and data privacy, documented in the institutional data protection concept of the Institute for Community Medicine [44]. Only the staff members in the examination center have access to personal data during baseline and follow-up examinations.

In addition, all participants receive a study ID card including an 1-D barcode at the baseline examination to support the correct assignment of the participants to their data over time.

### Data analysis

ActiLife software (version 6.13.2 or later, ActiGraph, Pensacola, FL, USA) will be used for data download and data processing. A valid measurement day is defined as a record of at least 10 h total wearing time and a record time of at least 4 days is required for data analysis. To categorize PA intensity, we used specific cut points based on Freedson [45]. PA is divided into sedentary (0–99 steps), light (100–1951 steps), moderate (1952–5724 steps), and vigorous (5725–9498 steps) PA. Step counts are based on 60-s epochs.

Statistical analysis will be conducted using pseudonymized data. First, the assessed project data will be descriptively analyzed. We will verify the conditions in both groups. In case of significant differences between the intervention and control group, analyses will be adapted accordingly and adjusted for relevant variables. Subsequently, the primary and secondary outcomes will be analyzed between the groups using *t* tests or corresponding non-parametric tests in case of ordinal variables or non-normality of the values. The tests will be conducted based on the intention-to-treat principle. The use of the intention-to-treat procedure ensures that each subject is included in the analysis. The analysis are carried out with the statistical software IBM SPSS Statistics (version 23.0.0.2 or later, IBM Corp., Armonk, NY, USA).

## Discussion

Previous studies have shown positive effects of interventions on PA and ST in older people using accelerometer devices. The interventions included using various different approaches, e.g., face-to-face consultations [24, 26] or regular telephone calls [46]. It seems to be difficult to transfer such complex procedures into routine practice due to the higher financial load and personnel expenses incurred.

On the basis of these considerations, our study was initiated to increase PA and reduce ST using a low-threshold intervention by providing automatically generated, individualized feedback letters containing health recommendations and personalized information about each participant’s PA and ST behavior. Over the course of the trial study participants twice receive individualized feedback based on accelerometer data. This method seems to be practicable to efficiently reach a high number of persons.

To promote PA in an adequate and lasting way, evidence on possible influencing factors is necessary. Therefore, the potential effect of self-efficacy on PA will be analyzed. Self-efficacy could be a relevant influence factor for changes in PA.

The results of the study will improve our understanding of PA and ST in older people and will provide evidence on how to increase the amount of PA and how to reduce ST behavior. If the intervention is effective, the study has the chance to reduce crucial risk factors for CVD and to improve quality of life, especially in the elderly.

### Trial status

The study started recruitment in November 2016, and is estimated to continue until the end of 2017. The final report will be prepared in 2018.

## Additional file

Additional file 1: SPIRIT 2013 Checklist. (DOC 120 kb)

## Abbreviations

BMBF: Federal Ministry of Education and Research
BZgA: Federal Centre for Health Education
CHD: Coronary heart disease
CVD: Cardiovascular diseases
DZHK: German Centre for Cardiovascular Research
eCRF: Electronic care report form
FGÖ: Fonds Gesundes Österreich
German PAQ: German Physical Activity Questionnaire
IPAQ: International Physical Activity Questionnaire
MOST: Measure of Older Adult’s Sedentary Time
MVPA: Moderate-to-vigorous physical activity
NCD: Non-communicable disease
NHANES: National Health and Nutrition Survey
PA: Physical activity
SF-8: Short Form 8
SOP: Standard operating procedures
ST: Sitting time
WHO: World Health Organization
